# Supplementary material for: A meta-analysis of neural systems underlying delay discounting: Implications for transdiagnostic research
Source: Imaging Neurosci (Camb). 2026 Mar 23;4:IMAG.a.1170. doi: 10.1162/IMAG.a.1170 (PMC13010365; doi:10.1162/IMAG.a.1170)
Supplement: Supplementary Material [file IMAG.a.1170_supp.pdf]

A meta-analysis of neural systems underlying delay discounting: Implications for transdiagnostic research

Nikhil V. Lakhani*, Min K. Souther*, Bema Boateng, Joseph W. Kable

Department of Psychology, University of Pennsylvania
* Equal contribution
Corresponding author: Joseph W. Kable (kable@psych.upenn.edu)

Supplementary Table S1. GingerALE meta-analysis cluster list.

	Voxel count	MNI coordinates[1] X	Y	Z	Max value[2]	Brain area
Task						
1	714	0	21	44	0.045	Bilateral dmPFC
2	274	37	20	-2	0.0376	R insula
3	270	-46	10	31	0.0357	L middle frontal gyrus
4	245	-33	21	0	0.0316	L insula
5	152	46	40	15	0.031	R frontal pole
6	308	-29	-57	47	0.029	L occipital cortex
7	123	-16	-93	-8	0.0269	L occipital pole
8	290	33	-61	46	0.0263	R occipital cortex
Hard						
9	455	3	24	43	0.0259	Bilateral dmPFC
10	175	-34	19	-2	0.0227	L insula
11	184	35	22	-4	0.0206	R insula
12	88	1	0	29	0.018	Bilateral anterior cingulate
Easy						
13	143	-62	-33	28	0.0212	L supramarginal gyrus
14	69	-58	-7	-10	0.0164	L superior temporal gyrus
15	85	5	-32	35	0.0143	R posterior cingulate
SV						
16	787	-2	8	-3	0.0636	Bilateral striatum
17	479	1	47	1	0.0346	Bilateral vmPFC
18	183	-63	-35	-4	0.0274	L middle temporal gyrus
19	185	-4	-40	34	0.0202	L posterior cingulate
Magnitude						
20	180	10	8	-5	0.0265	R striatum
Delay						
21	91	52	16	31	0.0193	R middle frontal gyrus
22	131	49	37	2	0.017	R frontal pole
23	98	-9	44	15	0.0152	L anterior cingulate
Immediacy						
24	136	-6	52	-6	0.0172	L vmPFC
SSR						
25	62	-4	55	25	0.0135	L dmPFC
LLR						
26	268	-37	-22	53	0.0344	L precentral gyrus
27	87	34	-78	-12	0.0238	R occipital fusiform gyrus
28	100	-23	-85	-10	0.0218	L occipital fusiform gyrus

[1] Coordinates correspond to cluster center of mass.
[2] Max value of ALE test statistic, i.e., probability that at least one focus of activation truly lies in a given location.
L = left; R = right; dmPFC = dorsomedial prefrontal cortex; vmPFC = ventromedial prefrontal cortex; SV = subjective value; SSR = smaller sooner reward; LLR = larger later reward.

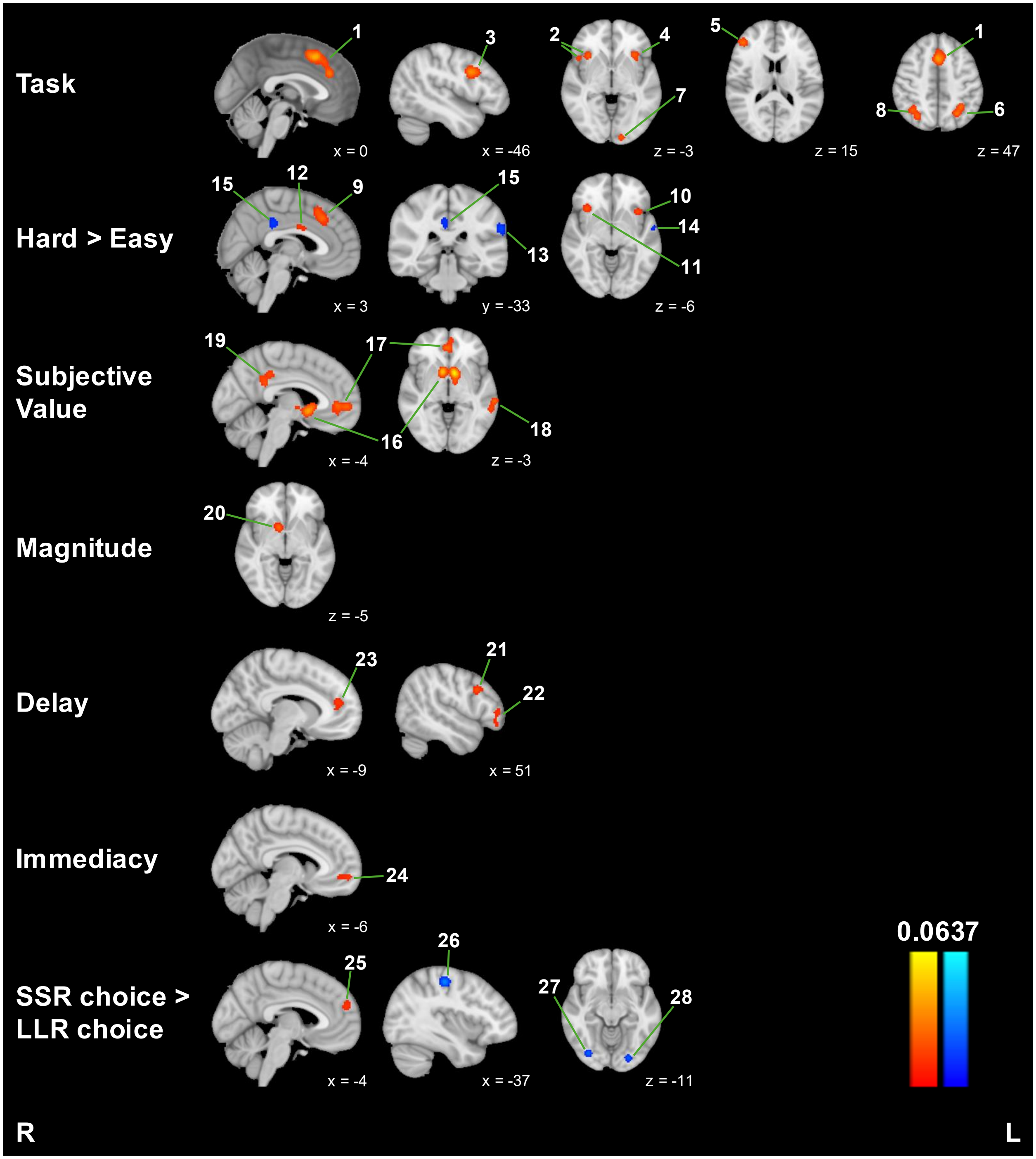

L = left; R = right; SSR = smaller sooner reward; LLR = larger later reward.

Supplementary Fig. S1. GingerALE meta-analysis results. Warm colors represent positive effects, and cool colors represent negative effects. Numbered labels correspond to cluster numbers in Supplementary Table S1.

Supplementary Table S2. MKDA meta-analysis cluster list, excluding studies with clinical populations.

	Volume (mm^3)	MNI coordinates[3] X	Y	Z	Max value[4]	Brain area
Task						
1	688	-2	15	50	57.9	Bilateral dmPFC
2	542	-29	-58	46	42.1	L occipital cortex
3	382	34	-59	45	36.8	R occipital cortex
4	344	-45	11	30	31.6	L middle frontal gyrus
5	334	-33	21	0	31.6	L insula
6	230	34	20	0	31.6	R insula
7	223	46	41	15	31.6	R frontal pole
Hard						
8	930	4	24	44	50	Bilateral dmPFC
Easy						
			No significant clusters			
SV						
9	1472	-3	6	-3	61.9	Bilateral striatum
10	1067	2	45	3	38.1	Bilateral vmPFC
11	312	-5	-40	35	33.3	L posterior cingulate
Magnitude						
12	505	10	9	-5	55.6	R striatum
Delay						
13	334	50	37	3	62.5	R frontal pole
14	454	-4	44	12	50	Bilateral anterior cingulate
15	387	51	15	31	50	R middle frontal gyrus
Immediacy						
16	1554	-4	49	2	50	Bilateral vmPFC / anterior cingulate
17	588	2	9	-3	37.5	Bilateral striatum
SSR						
18	444	-5	55	24	42.9	Bilateral dmPFC
LLR						
19	208	-36	-21	54	31.6	L precentral gyrus

[3] Coordinates correspond to cluster center of mass.
[4] Max value of MKDA test statistic, i.e., percentage of studies reporting a focus within 10mm from a given location.
L = left; R = right; dmPFC = dorsomedial prefrontal cortex; vmPFC = ventromedial prefrontal cortex; SV = subjective value; SSR = smaller sooner reward; LLR = larger later reward.

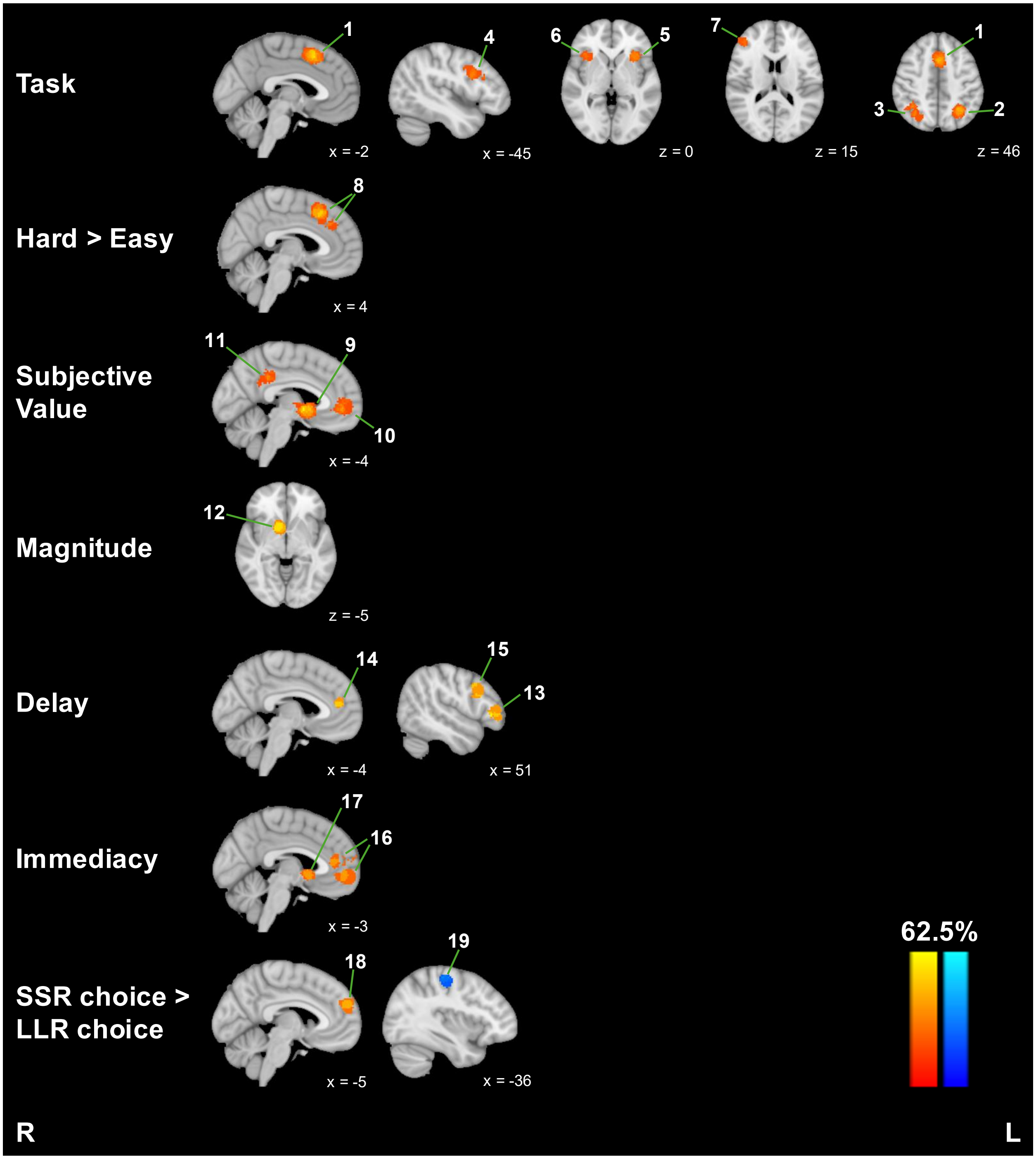

L = left; R = right; SSR = smaller sooner reward; LLR = larger later reward.

Supplementary Fig. S2. MKDA meta-analysis results, excluding studies with clinical populations. Warm colors represent positive effects, and cool colors represent negative effects. Numbered labels correspond to cluster numbers in Supplementary Table S2.

Supplementary Table S3. GingerALE meta-analysis cluster list, excluding studies with clinical populations.

	Voxel count	MNI coordinates[5] X	Y	Z	Max value[6]	Brain area
Task						
1	373	-2	15	50	0.0317	Bilateral dmPFC
2	287	-29	-58	47	0.028	L occipital cortex
3	206	-33	21	0	0.0246	L insula
4	110	34	20	1	0.023	R insula
5	97	47	41	16	0.0217	R frontal pole
6	141	-45	10	30	0.0201	L middle frontal gyrus
7	151	33	-62	45	0.0188	R occipital cortex
Hard						
8	169	5	21	47	0.0179	R dmPFC
9	133	34	23	-7	0.0166	R insula
10	113	44	-41	44	0.0151	R supramarginal gyrus
Easy						
11	87	4	15	-7	0.0154	R striatum
12	92	-62	-33	29	0.013	L supramarginal gyrus
13	76	4	-32	32	0.0125	R posterior cingulate
SV						
14	689	-2	7	-3	0.0497	Bilateral striatum
15	324	3	46	1	0.0226	Bilateral vmPFC
16	109	-54	-58	20	0.0205	L angular gyrus
17	100	-5	-41	35	0.0168	L posterior cingulate
Magnitude						
18	181	10	8	-5	0.0265	R striatum
Delay						
19	91	52	16	31	0.0193	R middle frontal gyrus
20	131	49	37	2	0.017	R frontal pole
21	98	-9	44	15	0.0152	L anterior cingulate
22	64	-9	12	-1	0.0143	L striatum
Immediacy						
23	160	-6	52	-6	0.0172	L vmPFC
SSR						
24	98	-4	55	25	0.0134	L dmPFC
LLR						
25	157	-35	-22	53	0.0204	L precentral gyrus

[5] Coordinates correspond to cluster center of mass.
[6] Max value of ALE test statistic, i.e., probability that at least one focus of activation truly lies in a given location.
L = left; R = right; dmPFC = dorsomedial prefrontal cortex; vmPFC = ventromedial prefrontal cortex; SV = subjective value; SSR = smaller sooner reward; LLR = larger later reward.

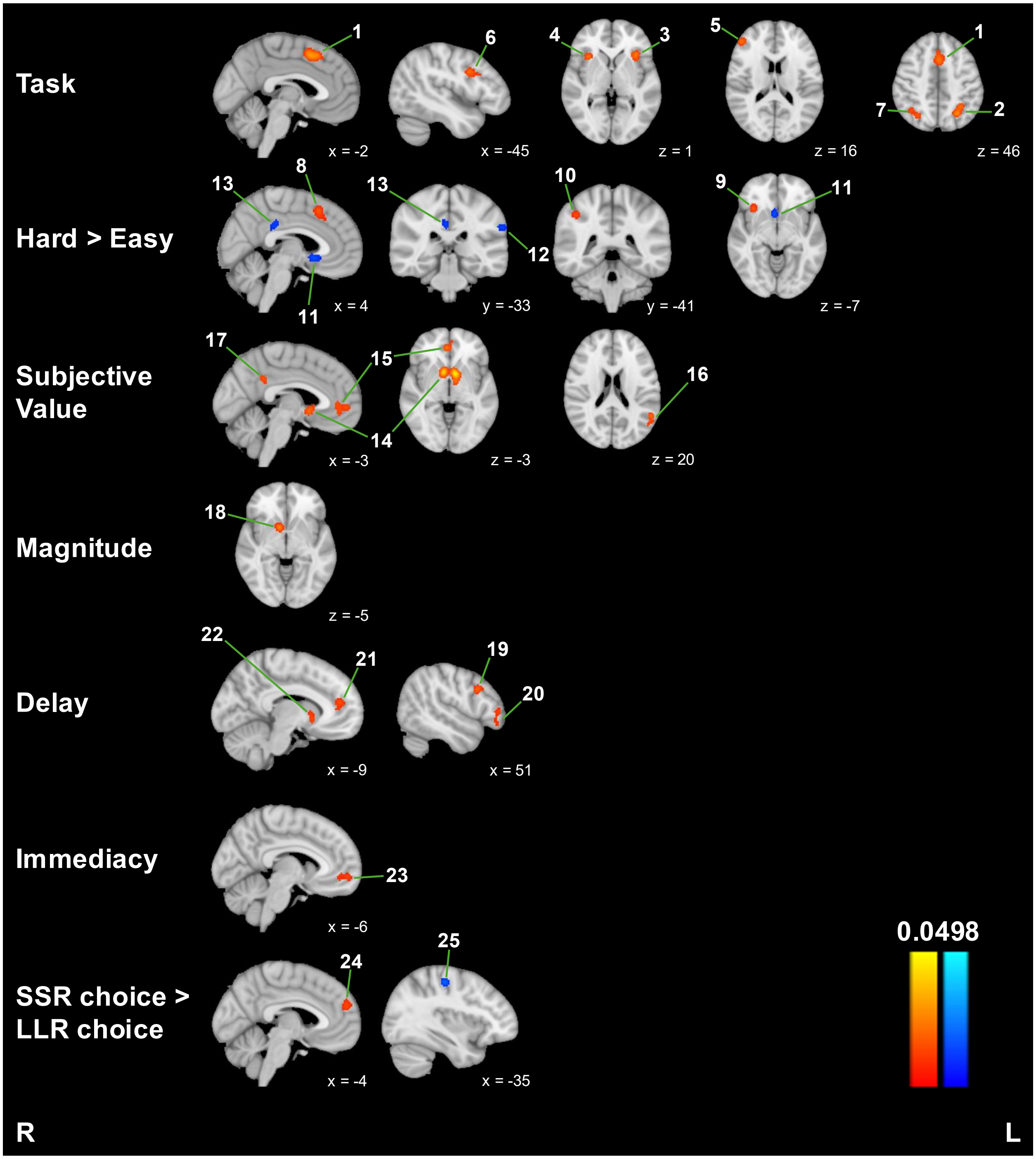

L = left; R = right; SSR = smaller sooner reward; LLR = larger later reward.

Supplementary Fig. S3. GingerALE meta-analysis results, excluding studies with clinical populations. Warm colors represent positive effects, and cool colors represent negative effects. Numbered labels correspond to cluster numbers in Supplementary Table S3.
